# Supplementary material for: Multifaceted Analyses of Isolated Mitochondria Establish the Anticancer Drug 2-Hydroxyoleic Acid as an Inhibitor of Substrate Oxidation and an Activator of Complex IV-Dependent State 3 Respiration
Source: Cells. 2022 Feb 7;11(3):578. doi: 10.3390/cells11030578 (PMC8834245; doi:10.3390/cells11030578)
Supplement: Supplementary file 1 [file cells-11-00578-s001.zip › Supplementary Material.pdf]

Supplementary Material: Details of LC-MS/MS Conducted for the Determination of DMH in Cell Lysates

Liquid chromatographic separation was obtained using a Cortecs (Waters) col-umn (C18, 2.7 µm particle size, 50 × 2.1 mm). The autosampler was set at 5 °C and the column was maintained at 40 °C during the entire analysis. Gradient elution mobile phases consisted of 10 mM ammonium acetate in water (phase A) and acetonitrile (phase B). Gradient elution (350 µL/min) increased linearly from 20% B towards 70% B in 2 min and maintained at 70% B for 3 min. DMH was detected in negative ion mode using electron spray ionization (ESI) and multiple reaction monitoring (MRM) mode of acquisition, using 21:0 Coenzyme A as internal standard. The TurboIonspray® probe temperature was set at 650 °C with the ion spray voltage at −4500V. The curtain gas was set at 40.0 psi. The nebulizer gas (Gas 1) was set to 70 psi, the turbo heater gas (Gas 2) was set to 70 psi and the collision gas (CAD) was set to 10 psi. The collision en-ergy (CE), declustering potential (DP) and collision cell exit potential (CXP) for the monitored transitions are given in Table S2. The dwell time was 50 ms. The levels in the samples were measured against standard curves.
