# Supplementary material for: Multifaceted Analyses of Isolated Mitochondria Establish the Anticancer Drug 2-Hydroxyoleic Acid as an Inhibitor of Substrate Oxidation and an Activator of Complex IV-Dependent State 3 Respiration
Source: Cells. 2022 Feb 7;11(3):578. doi: 10.3390/cells11030578 (PMC8834245; doi:10.3390/cells11030578)
Supplement: Supplementary file 1 [file cells-11-00578-s001.zip › TableS2.pdf]

Table S2: Monitored transitions in LC-MS/MS negative mode of DMH and the IS.

Compounds	Molecular ion $[M-2H]^{2-}/2$ (m/z)	Fragment (m/z)	DP (volts)	CE (volts)	CXP (volts)
Dimethylheptanoyl-CoA	452.6	408 (quantifier)	-120	-30	-15
	452.6	134 (qualifier)	-120	-34	-3
21:0 Coenzyme A	538.8	408 (quantifier)	-105	-34	-19
	538.8	134 (qualifier)	-105	-36	-7
